# The fluorescent pentameric oligothiophene pFTAA identifies filamentous tau in live neurons cultured from adult P301S tau mice

**DOI:** 10.3389/fnins.2015.00184

**Published:** 2015-05-29

**Authors:** Jack Brelstaff, Bernardino Ossola, Jonas J. Neher, Therése Klingstedt, K. Peter R. Nilsson, Michel Goedert, Maria Grazia Spillantini, Aviva M. Tolkovsky

**Affiliations:** ^1^Department of Clinical Neurosciences, University of CambridgeCambridge, UK; ^2^Department of Cellular Neurology, Hertie Institute for Clinical Brain Research, University of TübingenTübingen, Germany; ^3^Medical Research Council Laboratory of Molecular BiologyCambridge, UK; ^4^Department of Chemistry, Linköping UniversityLinköping, Sweden

**Keywords:** hyperphosphorylated tau, neurofibrillary tangles, transgenic P301S tau mouse, dorsal root ganglion neurons, Alzheimer's disease, frontotemporal dementia (FTD), fluorescent vital fibrillar tau dye

## Abstract

Identification of fluorescent dyes that label the filamentous protein aggregates characteristic of neurodegenerative disease, such as β-amyloid and tau in Alzheimer's disease, in a live cell culture system has previously been a major hurdle. Here we show that pentameric formyl thiophene acetic acid (pFTAA) fulfills this function in living neurons cultured from adult P301S tau transgenic mice. Injection of pFTAA into 5-month-old P301S tau mice detected cortical and DRG neurons immunoreactive for AT100, an antibody that identifies solely filamentous tau, or MC1, an antibody that identifies a conformational change in tau that is commensurate with neurofibrillary tangle formation in Alzheimer's disease brains. In fixed cultures of dorsal root ganglion (DRG) neurons, pFTAA binding, which also identified AT100 or MC1+ve neurons, followed a single, saturable binding curve with a half saturation constant of 0.14 μM, the first reported measurement of a binding affinity of a beta-sheet reactive dye to primary neurons harboring filamentous tau. Treatment with formic acid, which solubilizes filamentous tau, extracted pFTAA, and prevented the re-binding of pFTAA and MC1 without perturbing expression of soluble tau, detected using an anti-human tau (HT7) antibody. In live cultures, pFTAA only identified DRG neurons that, after fixation, were AT100/MC1+ve, confirming that these forms of tau pre-exist in live neurons. The utility of pFTAA to discriminate between living neurons containing filamentous tau from other neurons is demonstrated by showing that more pFTAA+ve neurons die than pFTAA-ve neurons over 25 days. Since pFTAA identifies fibrillar tau and other misfolded proteins in living neurons in culture and in animal models of several neurodegenerative diseases, as well as in human brains, it will have considerable application in sorting out disease mechanisms and in identifying disease-modifying drugs that will ultimately help establish the mechanisms of neurodegeneration in human neurodegenerative diseases.

## Introduction

Tau is a soluble microtubule-associated protein with dynamic phospho-epitopes that is expressed throughout the CNS and PNS (Cleveland et al., 1977; Binder et al., 1985). Its primary role is the stabilization of microtubules thereby facilitating axonal transport and maintaining proper neuronal morphology (Mandelkow and Mandelkow, 2012). However, insoluble hyperphosphorylated filamentous tau forms the neurofibrillary tangles (NFTs) and Pick bodies that are diagnostic hallmarks of numerous neurodegenerative diseases including Alzheimer's disease (AD), Pick's disease, frontotemporal dementia with Parkinsonism linked to chromosome 17, progressive supranuclear palsy and cortical basal degeneration (Spillantini and Goedert, 2013). Indeed, the *MAPT* (tau) gene haplotype H1/H1 is also associated with memory dysfunction in patients with Parkinson's disease (Winder-Rhodes et al., 2015) and acts as an independent genetic risk factor in pathologically proven PD (Charlesworth et al., 2012). Moreover, abnormal NFTs of tau were also found in the brains of Huntington's disease patients (Fernandez-Nogales et al., 2014; Vuono et al., 2015). The driving force behind tau aggregation is not known but familial mutations in the MAPT gene such as P301S can increase aggregation propensity by reducing tau binding to microtubules, and possibly by introducing a new phosphorylation site (Hong et al., 1998). Individuals affected by this mutation have an early to midlife age of onset and an aggressive disease progression (Bugiani et al., 1999; Sperfeld et al., 1999; Yasuda et al., 2000; Lossos et al., 2003).

Although both beta-amyloid and tau form abnormal filaments in AD, there is increasing evidence suggesting that tau is necessary for mediating the ultimate neurodegeneration (Pooler et al., 2013; Bloom, 2014). However, despite extensive research into the mechanisms of degeneration, it is still unclear how tau mediates its toxic effects neither in pure tauopathies, nor in the context of beta amyloid plaques in AD. There is a continued debate as to whether tau+ve NFTs induce cell death (Tomlinson et al., 1970; Gomez-Isla et al., 1997; Mocanu et al., 2008; Fatouros et al., 2012) or whether protein aggregates are benign (Kuchibhotla et al., 2014) or even protective (Morsch et al., 1999; Spires et al., 2006; Fox et al., 2011). Perhaps a “one hit” model, which predicts that abnormally misfolded tau is both necessary and sufficient for induction of toxicity, as was suggested to account for the progression of several neurodegenerative diseases (Clarke et al., 2000, modified by Clarke and Lumsden, 2005), needs to be replaced by a “two hit” model, where another event, which is not toxic *per se*, is required for tau to cause cell death. The second hit could come from a myriad of sources, which might also explain why it has been so difficult to demonstrate a clear single mechanism of cell death in AD, unlike the more defined cases of regulated cell death such as apoptosis during development (Dekkers and Barde, 2013; Kole et al., 2013), which fit a modified one hit model (Edwards and Tolkovsky, 1994), or parthanatos (Fatokun et al., 2014).

To study whether cell autonomous mechanisms lead to tau pathology and neurodegeneration, we turned to the transgenic P301S tau mouse model, which develops progressive hallmarks of tau pathology that culminate in neuronal cell death over 5 months. We showed previously that a subset of Dorsal Root Ganglion (DRG) neurons from transgenic human P301S tau mice develop progressive hallmarks of tau pathology *in vivo* and *in vitro* with the same time course as that of CNS neurons (Allen et al., 2002; Mellone et al., 2013). These neurons are unique in that they can be cultured from adult mice for months, enabling tau-dependent processes to be defined as tau evolves from a soluble to a filamentous form with a characteristic acquisition of different hyperphosphorylation and conformational patterns (Mellone et al., 2013). Nevertheless, despite the presence of sarkosyl-insoluble filamentous tau in DRG extracts and evidence for conformational changes associated with filamentous tau in cultured DRG neurons (Mellone et al., 2013), supported, for example, by staining with the conformation-specific antibody MC1 (Jicha et al., 1997, 1999), there has been no direct evidence that tau adopts these conformations in living DRG neurons. This is most likely due to the low intensity of specific signals elicited by beta-sheet-reactive dyes such as thioflavins (Allen et al., 2002) or FSB (Velasco et al., 2008) in the context of the large volumes of the proprioceptive and mechanoceptive neurons that express P301S tau in the transgenic model. The ability to visualize filamentous tau aggregates in specific cells within a heterogeneous culture would allow investigations of the cell autonomous processes leading to toxicity or protection.

Recently a new family of luminescent conjugated polythiophene dyes were described that detect various beta-sheet containing proteins. Some members of this family are capable of discriminating between different conformational strains of PrP^Sc^, the prion disease associated aggregate form of normal prion protein PrP^C^, that would be indistinguishable by immunohistochemistry alone (Sigurdson et al., 2007), while others can detect beta amyloid and tau filaments in post-mortem AD brains (Aslund et al., 2009). In particular, the pentameric formyl thiophene acetic acid (pFTAA) is a highly promising compound, as it can be used to distinguish different conformations of beta-sheet containing proteins due to specific conformation-dependent shifts in emission spectra depending on the nature of the protein (Klingstedt et al., 2013). Intravenous injection of pFTAA into APP23 mice was reported to produce no discernable toxic effects on body weight, whole blood cell counts, or histology of peripheral organs (Wegenast-Braun et al., 2012). In AD brains, pFTAA co-labeled neurons immunostained for AT8, an antibody that correlates with tau pathology (Aslund et al., 2009), but given that AT8 is not specific to insoluble filamentous tau aggregates, more complete characterization with markers of filamentous tau was lacking. The clarity of staining of neurons in a brain stem section from 6-month-old P301S tau transgenic mice (Klingstedt et al., 2013), a stage at which there is considerable accumulation of filamentous tau in cortical and motor neurons in the CNS and spinal cord, and the lack of apparent toxicity, prompted us to investigate whether we could use pFTAA to identify living DRG neurons that express filamentous tau. Here we show that pFTAA binds filamentous tau aggregates in DRG neurons of P301S tau mice by co-labeling with antibodies that detect aggregated/filamentous tau. Moreover, we show that both live and fixed cultured DRG neurons that are immunoreactive for these antibodies co-label with pFTAA. Importantly, we show that we can follow the same fields of living pFTAA+ve neurons in long-term cultures and quantify their death, an observation that will allow us to underpin the mechanism of toxicity and identify drugs that may alleviate it.

## Materials and methods

### Animals

Homozygous mice transgenic for P301S tau (Allen et al., 2002), or wild-type human 2N4R tau (Alz17) (Probst et al., 2000; Clavaguera et al., 2009), and C57BL/6S (C57BL/6 OlaHsd; Harlan) control mice were maintained as described previously (Mellone et al., 2013). All procedures were performed in accordance with the UK Home Office Regulations for the Care and Use of Laboratory Animals and the UK Animals (Scientific Procedures) Act 1986 and were approved by the Cambridge University local ethical committee. To label neurons *in vivo*, 150 nmole of pFTAA in 150 μl saline were injected intravenously via the tail vein. After 48 h, the mouse was perfused transcardially with PBS followed by 4% paraformaldehyde in PBS. Brains and DRGs were removed and treated as described below.

### Cultures

DRG neurons were cultured on 13 mm coverslips or glass-bottom dishes (MatTek) coated with poly-D-lysine and laminin and maintained as described previously (Mellone et al., 2013). For live labeling experiments, neurons were cultured for 7 days in growth medium containing fluorodeoxyuridine (to remove non-neuronal cells and neurons that die due to injury during preparation), then washed and labeled in medium containing 3 μM pFTAA and 0.1 μg/ml propidium iodide (PI) for 30 min at room temperature. Medium containing PI was replaced every 3 days but no new pFTAA was added to ensure that no new pathological tau assemblies forming over time as the culture progressed were counted.

### Immunocytochemistry

The brain and DRGs removed from P301S tau mice after perfusion were incubated overnight in 4% PFA, washed and stored in 30% sucrose in PBS (brain) or PBS containing 0.1% sodium azide (DRG). Brain was cut into 30 μm sections on a sliding microtome (Leica SM2000 R). Prior to antibody staining, brain sections or DRG were either immersed in acetone for 10 min (Nilsson et al., 2012), and then rehydrated in water followed by PBS without detergent, or incubated in PBS containing 0.3% triton X-100. There was no difference in the staining intensity or pattern between the two procedures. To label whole DRG *ex vivo*, DRG were isolated from 5-month-old P301S tau mice or C57BL/6 OlaHsd (Harlan) mice and incubated with 15 μM pFTAA in DMEM medium containing 20 ng/ml NGF and 1% fetal bovine serum at 4°C for 15 h, then fixed in 4% PFA for 20 min at room temperature. Following studies of the time course and dose-dependence to establish the best conditions for pFTAA labeling of tau, it was determined that an incubation of 30 min with 3 μM pFTAA was sufficient to label filamentous tau+ve DRG neurons to the same extent as higher concentrations and longer times. Before labeling, ganglia were permeabilised by incubating with acetone or PBS with 0.3% triton X-100 as described for the brain sections. Ganglia were bisected with a scalpel blade to facilitate antibody penetration. Antibodies were either added in PBS (if acetone was used) or PBS with 0.3% triton X-100.

Dissociated DRG neurons were fixed on ice with 95% ethanol kept at −20°C, then rehydrated through 70% and 50% alcohol and water before storage at 4°C in PBS with 0.1% azide. Ice-cold methanol (100%) was equally effective at fixation. pFTAA diluted in PBS was added to fixed neurons or in growth medium when added to living neurons. Neurons were washed in PBS before the addition of primary antibodies. Incubation in primary antibody solution was at 4°C over night for cultured neurons, or 2 days at 4°C for brain sections or whole DRG to facilitate antibody penetration into the tissue. After washes in PBS, secondary antibody was applied for 1 h (cultures) or 1 day (brain sections, whole DRG) in the same buffers used for primary antibodies. Brain sections or cultures on coverslips were mounted in FluorSave™ (Calbiochem).

### Antibodies

Anti-phospho-tau AT100 (epitope pT212/pS214, MN1060) and anti-human tau HT7 (epitope 159–163, MN1000) mouse monoclonal antibodies were from Thermo Scientific and used at 1:1000 or 1:500 dilution, respectively. Hybridoma culture supernatant containing MC1 was a kind gift from Dr. P Davies, Albert Einstein College of Medicine, New York and used at 1:500-1:1000 dilution. Anti-β-III tubulin antibody was from Covance (PRB-435P-100) and used at 1:1000 dilution. AlexaFluor®568/647/350 conjugated secondary antibodies (Invitrogen) appropriate for the species were used at 1:1000 dilution. pFTAA was excited using the L5 filter (Leica). No significant fluorescent emission of pFTAA bound to tau was detected upon excitation at 540/50 nm, the wavelength used to excite AlexaFluor®568.

### Imaging and data analysis

Imaging was performed using a Leica DMI 4000 B microscope and Leica-LAF software. Images were exported in TIFF format and figures compiled using Adobe Photoshop CS3. Quantification of concordance rates between pFTAA+ve neurons and co-stains was achieved by imaging 30 random fields at 20x magnification across 3 biological replicates, yielding 133–224 pFTAA+ve cells. Subsequent analysis used ImageJ (Rasband, W.S., ImageJ, U. S. National Institutes of Health, Bethesda, Maryland, USA, http://imagej.nih.gov/ij/, 1997–2014). Sampling for calculation of the half saturation constant was achieved by imaging 15 randomly chosen pFTAA+ve cells per concentration. Relative fluorescence intensity of each pFTAA+ve neuron per concentration was quantified using the ImageJ Measure tool. Microscope settings were: filter cube L5, intensity 5, exposure 170 ms, gain 1. The half saturation constant and the coefficient of determination (R^2^) values were determined using the non-linear least squares curve-fitting program http://faculty.gvsu.edu/carlsont/232lab/nonlin2.html. To determine the rate of cell death, the number of pFTAA+ve cells that became PI positive and/or were subsequently lost from the culture, were expressed as a percent of total pFTAA+ve neurons imaged at *t* = 0 in 3 biological replicates. Significance of changes were calculated by one sample *t*-test. The number of pFTAA-ve neurons was identified by phase contrast morphology in the same fields as those used to image pFTAA+ve neurons, and were counted at onset of the experiment and after 25 days *in vitro*. Significance of difference between the percentage of pFTAA+ve and pFTAA-ve neurons remaining in the dish was determined by a paired *t*-test.

## Results

**Figure 1:**
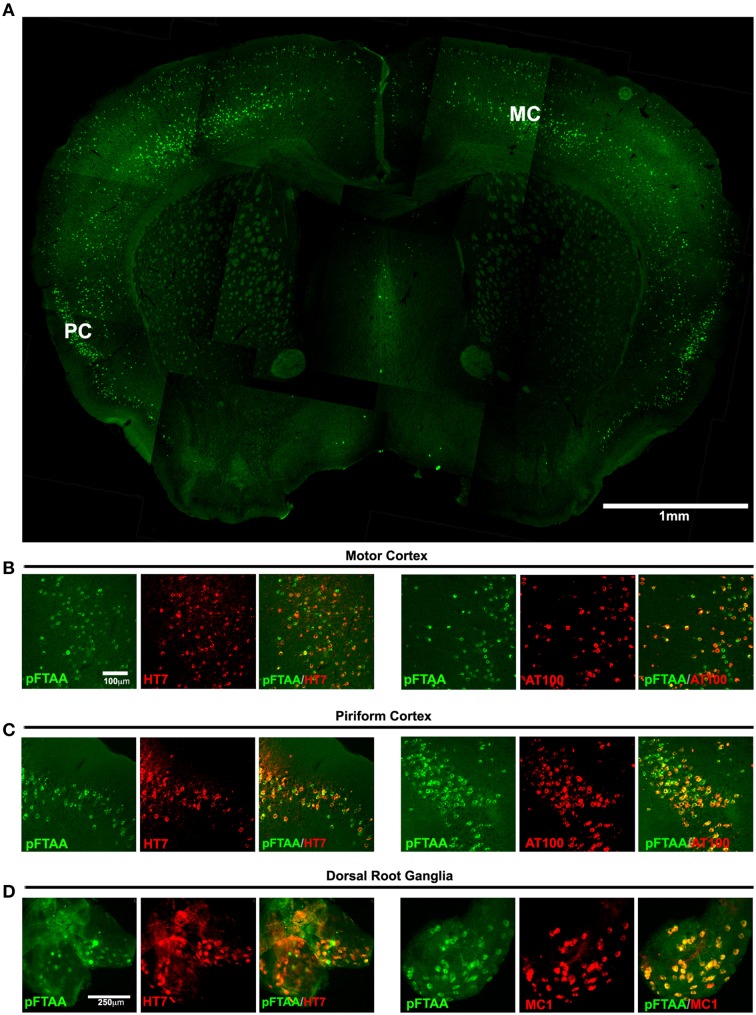
**Labeling of tau inclusions in the brain and DRG of 5-month-old P301S transgenic mouse by pFTAA**. A P301S tau transgenic mouse injected with 150 nmole pFTAA via the tail vein was perfused after 48 h. **(A)** A composite image of a 30 μm coronal brain section at the level of the anterior commissure (5x objective) showing the general staining pattern of pFTAA+ve neurons; staining is found almost exclusively in the cortex. MC, motor M1 cortex, PC, piriform cortex. **(B)** motor M1 cortex and **(C)** piriform cortex stained with pFTAA (green) and immunolabeled with anti-human tau antibody HT7, or anti-fibrillar phospho-tau-specific antibody AT100 (red). **(D)** whole mount of a DRG stained with pFTAA *in vivo* (green) and immunolabeled with anti-human tau antibody HT7, or the conformational anti-tau antibody MC1 (red). Note that due to poorer penetration of the antibodies compared to pFTAA, some pFTAA+ve neurons that appear HT7-ve at the exposure shown are co-stained by the antibodies upon increased exposure. Scale bar, 1 mm **(A)** 100 μm **(B,C)**, 250 μm **(D)**.

We first examined whether DRG neurons in P301S tau transgenic mice—like CNS neurons (Klingstedt et al., 2013)—were labeled after injection of pFTAA *in vivo*, and whether pFTAA-labeled neurons co-label with antibodies that detect insoluble tau aggregates and fibrils. Figure 1A shows an overview of a brain section taken from the level of the anterior commissure; pFTAA labeled numerous neurons in the upper and deeper layers of the motor cortex, and most of the neurons in the piriform cortex, but none in the striatum. This labeling occurred exclusively in P301S tau+ve neurons, detected with the human-specific tau antibody HT7 (Figures 1B–D), and coincided with positive immunolabeling with the anti-phospho (pT212/pS214) tau antibody AT100 (Figures 1B,C), which only recognizes sarkosyl-insoluble, but not soluble, tau in the CNS of P301S tau mice (Delobel et al., 2008). Due to comparatively poorer antibody penetration compared to pFTAA, some structures that first appeared HT7- or AT100-negative, were found to be positively stained after increasing the camera exposure time. Colocalization was also obtained after immunolabeling with the anti-phospho (pSer202/pThr205) tau antibody AT8, which binds to filamentous tau but also binds to soluble hyperphosphorylated tau (Delobel et al., 2008) (data not shown). A subset of neurons in the DRG were also labeled with pFTAA and, as in the brain, this labeling coincided with immunolabeling with HT7 or the MC1 antibody (Figure 1D), which detects a conformational change in tau that is associated with neurofibrillary tangles in human AD brains (Weaver et al., 2000) and is only evident in DRG neurons from P301S tau mice when they are also AT100+ve (Mellone et al., 2013). In biochemical studies, MC1 was shown to bind with the highest affinity to a compacted “paper clip” form of misfolded tau when the protein is pseudo-phosphorylated at the three epitopes recognized by the antibody PHF-1 [which stains most of the HT7+ve DRG neurons from P301S tau mice (Mellone et al., 2013)], AT8 and AT100 antibodies (Jeganathan et al., 2012). It should be noted that only 7–20% of DRG neurons are P301S tau (HT7)+ve in ganglia from 5-month-old P301S tau mice (Mellone et al., 2013).

**Figure 2:**
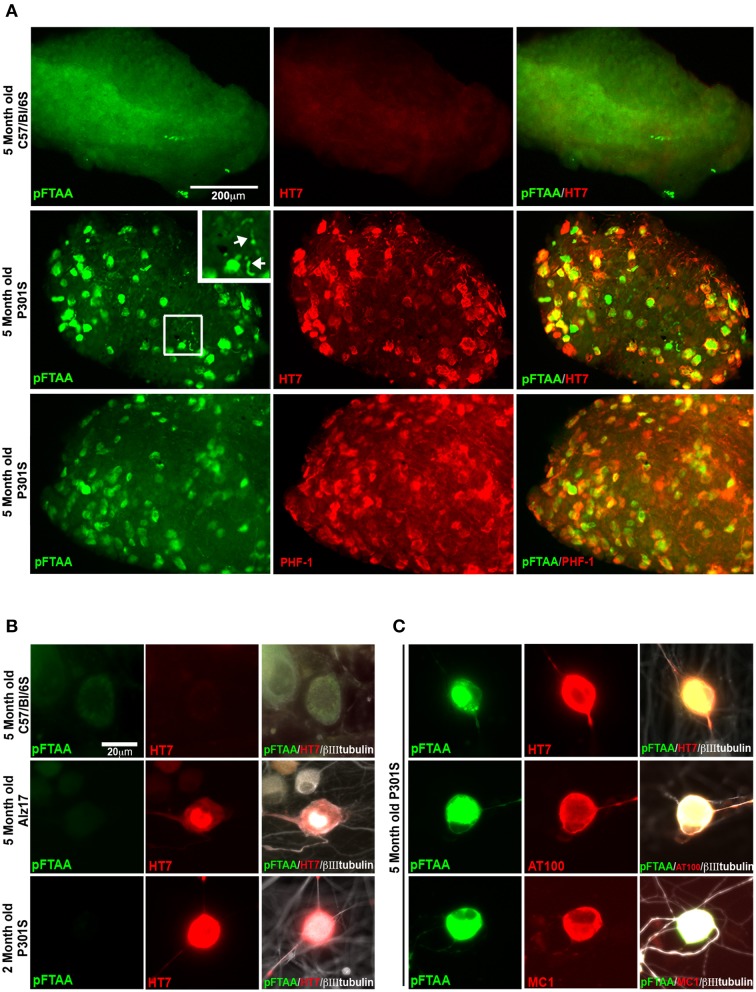
**pFTAA selectively labels fibrillar tau in DRG neurons in intact DRG *ex vivo* and cultured dissociated DRG neurons from 5-month-old P301S tau transgenic mice. (A)** Intact DRG from 5-month-old C57BL/6 OlaHsd (wt, top row) or P301S tau mice (middle row) were incubated with 15 μM pFTAA at 4°C for 15 h, then fixed in 95% ethanol, rehydrated, and immunolabeling (red) with the HT7 antibody. Bottom row shows immunolabeling with the PHF-1 antibody (which recognizes phospho-serines396/404). Magnification box identifies pFTAA+ve threads visible in the axons of whole ganglia (arrows). Non-specific signal in wt intact ganglia is a-cellular debris carried over because of re-use of the dye. **(B)** DRG neurons were cultured from 5-month-old C57BL/6 OlaHsd wild type mice, or Alz17 mice transgenic for the longest isoform of human tau (2N4R), or 2-month-old P301S tau mice for 2 days. After ethanol fixation, cultures were labeled with pFTAA and immunolabeled with HT7. Note absence of pFTAA staining but presence of human tau in the neurons from Alz17 and 2-month-old P301S tau mice. (**C**) Dissociated DRG neurons from 5-month-old P301S tau mice were labeled after ethanol fixation by pFTAA (green), as well as for AT100 or MC1 (red). Images are representative of 133–224 pFTAA+ve cells across 3 biological replicates. Scale bar, 200 μm in **(A)** 20 μm in **(B,C)**.

To investigate whether pFTAA also labels specific DRG neurons in whole ganglia *ex vivo*, whole DRG were excised from 5-month-old P301S tau mice or C57BL/6S wild type (wt) mice, labeled with pFTAA, then fixed and immunostained. Figure 2A shows that pFTAA did not label any DRG neurons in ganglia from wt mice but did label the same subset of neurons in DRG from P301S tau mice that were also immunolabeled with HT7. Because the serine 396/404 phospho epitope recognized by PHF-1 is implicated in high affinity binding of MC1 to tau fibrils, we also stained the DRG for PHF-1. Figure 2A (bottom row) shows that there was extensive co-localization of pFTAA and PHF-1+ve neurons.

To establish whether pFTAA labeled cultured DRG neurons, and determine whether this labeling was specific to P301S tau+ve cells expressing advanced forms of tau pathology, DRG neurons were cultured from 5-month-old wt C57BL/6S mice, or Alz17 mice, which express the 2N4R isoform of human tau under the same Thy1.2 promoter encoding a neuron-restricted element as that used to express P301S tau (Probst et al., 2000; Clavaguera et al., 2009), and 2- or 5-month-old P301S tau mice. After 2 days, cultures were fixed, and stained with pFTAA. Figure 2B shows that no staining of pFTAA was detected in wt DRG neurons, nor in HT7+ve neurons from Alz17 mice, which do not express filamentous tau aggregates at this age, nor in HT7+ve neurons from 2-month-old P301S tau mice, a stage when they express P301S tau protein but do not form insoluble tau fibrils (Delobel et al., 2008). By contrast, pFTAA stained 44.1 ± 0.9% of HT7+ve, 81.0 ± 11.4% of AT100+ve, and 87.3 ± 4.3% of MC1+ve neurons (mean ± SD, 3 independent cultures) cultured from 5-month-old P301S tau mice (Figure 2C). The lower percentage of pFTAA staining of HT7+ve neurons compared to AT100 or MC1+ve neurons reflects the fact that only ~40% of HT7+ve neurons taken from this stage have developed the advanced stages of tau aggregation that is detected by the AT100 and MC1 antibodies (Mellone et al., 2013).

**Figure 3:**
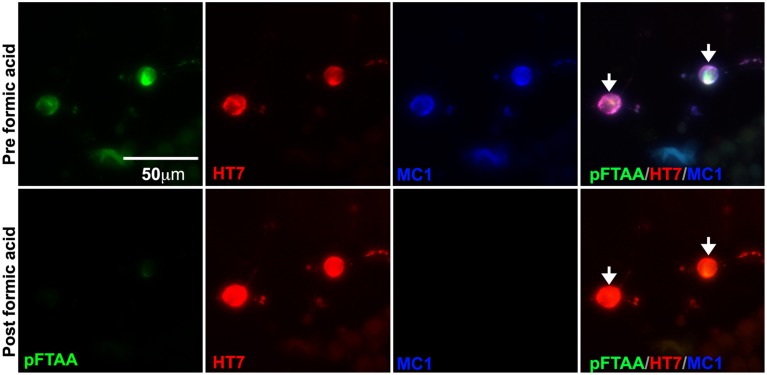
**Formic acid treatment solubilizes fibrillar tau, but not souble tau, and eliminates the binding of pFTAA**. DRG neurons cultured from 5-month-old P301S tau mice for 2 days were fixed with 95% ethanol. Top row shows pFTAA+ve neurons immunolabeled with HT7 (red) and MC1 (blue) antibodies. Bottom row shows the same set of neurons re-stained with pFTAA and antibodies after a 15 min treatment with 90% formic acid. Note that formic acid treatment prevented the re-binding of pFTAA and the MC1 antibody whereas the interaction with HT7, which binds to soluble and insoluble tau, was restored. Scale bar, 50 μm.

To further verify that pFTAA was binding to insoluble filamentous tau, cultured DRG neurons were imaged before and after formic acid treatment, which solubilizes filamentous tau in brain sections from Alzheimer's disease patients (Bing et al., 2006) and in brain sections from P301S tau mice (Velasco et al., 2008). DRG neurons were fixed, stained with pFTAA, and immunolabeled with MC1 and HT7 antibodies. Cells were imaged, then treated with 90% formic acid for 15 min after which the same cells were re-stained with pFTAA, re-probed with the indicated antibodies, and re-imaged. Figure 3 shows that no pFTAA or MC1 staining was detected after incubation with formic acid, whereas HT7 immunostaining was still strongly visible. Hence, the pFTAA binding site is present only in insoluble tau with a pathological conformation.

**Figure 4:**
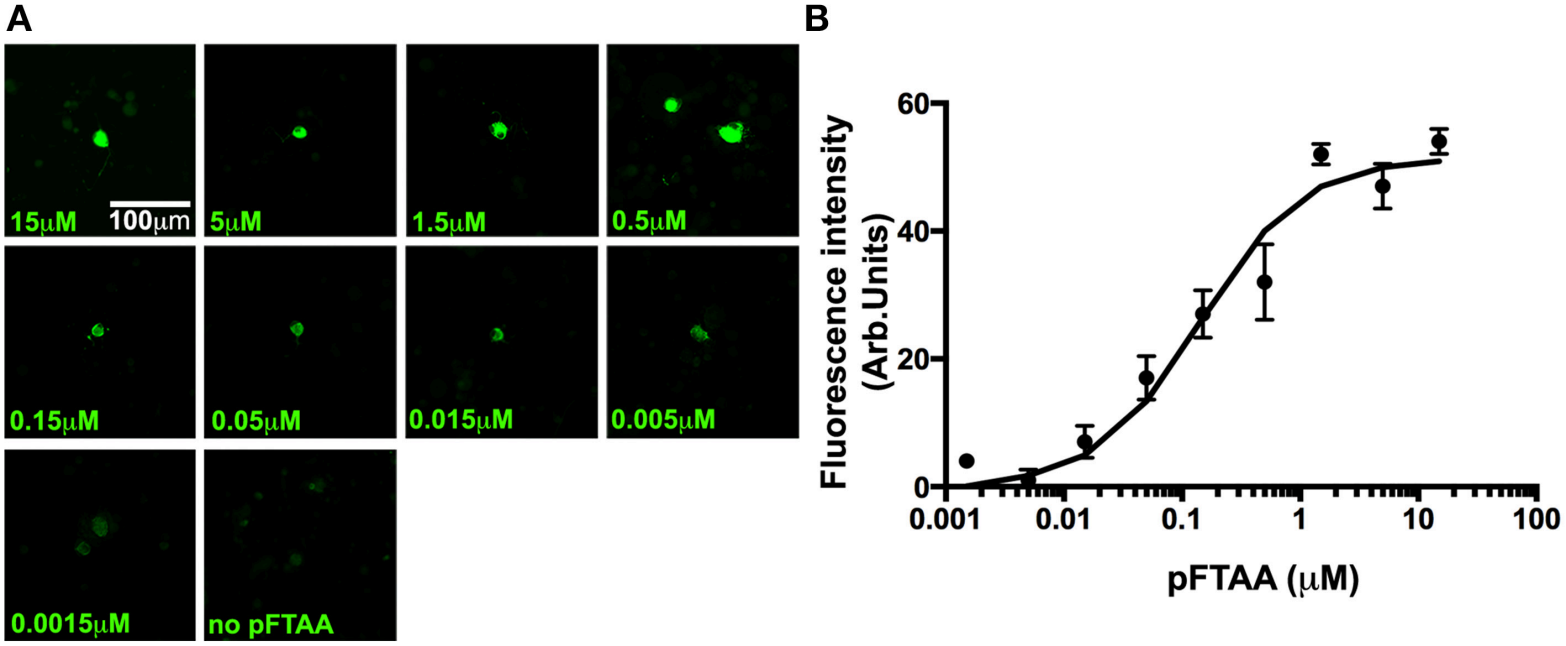
**A dose response of pFTAA demonstrates a single, homogeneous binding site to neurons expressing filamentous tau**. pFTAA was added at the concentrations indicated to ethanol-fixed DRG neurons cultured from 5-month-old P301S tau mice for 2 days. **(A)** Representative images of pFTAA binding intensities at the concentrations indicated. **(B)** Quantification of signal intensity. To determine the relationship between the concentration of pFTAA and fluorescence intensity, the fluorescence intensity values from 15 neurons per dose were averaged (circles) and the results were submitted to a least square curve fitting analysis according to an equation that predicts a single, saturable binding site (line) from which a half saturation constant of 0.142 μM (*R*^2^ = 0.983) was derived. Error bars indicate sem values. Scale bar, 100 μm.

The high signal to noise ratio of the pFTAA staining pattern prompted us to run a dose response curve to determine the relative affinity of pFTAA to P301S tau in the neurons. For this purpose, DRG neurons from 5-month-old P301S tau mice were cultured for 2 days, fixed, and stained with varying concentrations (0.0015–15 μM) of pFTAA. Figure 4A shows representative images of neurons stained with the different concentrations of pFTAA, while the average relative fluorescence intensity measurements from 15 random neurons per concentration are plotted in Figure 4B. To determine the half saturation constant (EC50), the data were fitted to an equation that assumes a single, saturable binding site (Fl_max_x [pFTAA])/(EC50 + [pFTAA]) by non-linear curve fitting, from which an EC50 value of 0.142 μM was calculated with a correlation coefficient of 0.983 over the entire fit. Such a high affinity is unique to pFTAA as it was impossible to resolve any specific binding using FSB or thioflavin T/S at similar concentrations.

**Figure 5:**
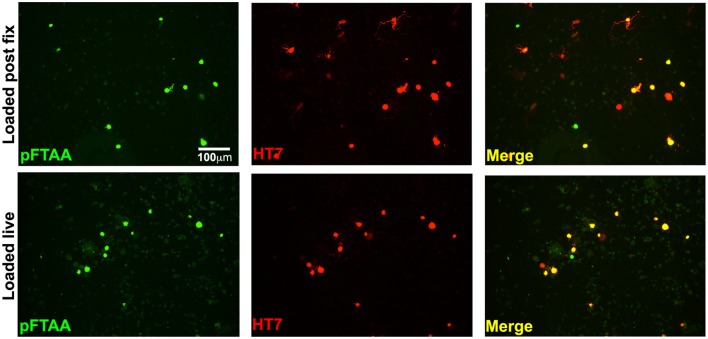
**Labeling of insoluble tau is not an artifact of fixation**. DRG neurons cultured from 5-month-old P301S tau mice for 2 days were either incubated with 3 μM pFTAA for 30 min at room temperature before fixation in 95% ethanol, or fixed before labeling with pFTAA using the same solution and conditions. Cultures were immunolabeled with the HT7 antibody (red). Loading of pFTAA into either live or fixed dissociated DRG neurons produced equivalent signal and co-localization with HT7. Note that some HT7+ve neurons are not labeled with pFTAA because HT7 neurons with insoluble tau only represent about 44% of all the HT7+ve neurons. Scale bar, 100 μm.

To examine whether live DRG neurons expressing insoluble tau would be detected with pFTAA, and whether it was the fixation that induced a conformation that allowed the binding of pFTAA to P301S tau, 2-day cultures of DRG neurons from 5-month-old mice were incubated with 3 μM pFTAA (rather than the higher concentration used in fixed cultures in order to minimize background fluorescence) for 30 min and then fixed, or fixed first and then stained with the same pFTAA solution. Figure 5 shows that pFTAA stained similar profiles of HT7+ve neurons in live cultures before fixation as those that were fixed prior to staining. Hence, the conformation of tau detected by pFTAA is preformed in the neurons and is not induced after – or by – fixation, as also demonstrated after delivering pFTAA *in vivo* or in ganglia stained by pFTAA before fixation.

**Figure 6:**
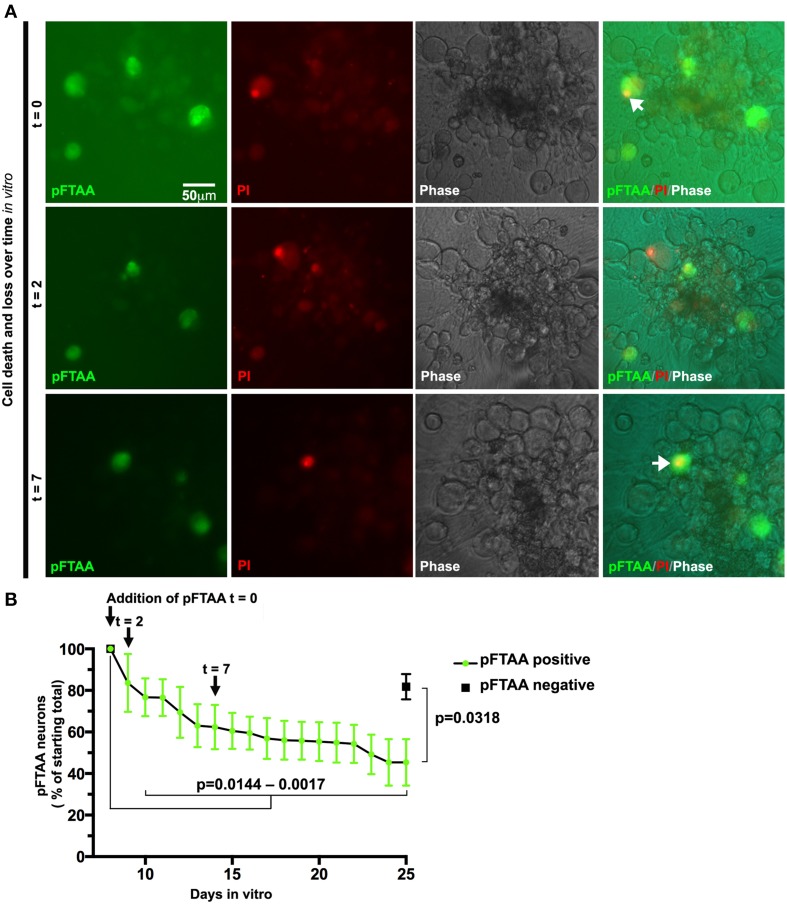
**Slow death of defined DRG neurons expressing fibrillar tau can be followed continuously after labeling with pFTAA**. DRG neurons were cultured from 5-month-old P301S tau mice for 7 days before labeling with 3 μM pFTAA, (t = time in days after addition of pFTAA). Medium was replaced with PI-containing medium without pFTAA every 3 days. **(A)** Images of the same set of neurons at 0, 2, and 7 days after addition of pFTAA showing appearance of PI+ve neurons and their subsequent disappearance from the cultures. **(B)** The loss of pFTAA+ve neurons as a percentage of total neurons originally labeled is 45% after 25 days *in vitro*. The loss of pFTAA+ve neurons becomes significant at 10 days *in vitro* (*p* = 0.0144) and continues to increase until 25 days *in vitro* (*p* = 0.0017). Black square denotes percentage survival of pFTAA-ve neurons in the same culture after 25 days (mean ± sem from the three biological replicates, *p* = 0.0318, paired *t*-test). Scale bar, 50 μm.

Prior to the use of pFTAA, neurons had to be fixed and stained with antibodies to detect those neurons containing advanced forms of aggregated tau. This meant that the same field of live neurons that had been studied, for example, to follow mitochondrial transport, had to be retraced after fixation and antibody staining (Mellone et al., 2013). Given the positive detection of neurons containing fibrillar tau using pFTAA, we asked whether we could visualize the progression of processes that occur in these neurons over longer periods of time. One such process of interest is the mechanism of cell death. As mentioned above, there is an ongoing debate as to tau+ve neurofibrillary tangles induce cell death or whether these protein aggregates are benign or even protective. We reported the loss of AT8 and AT100+ve neurons in long-term cultures of DRG neurons from P301S tau mice, but could not establish whether this was due to intrinsic properties or to do with post-fixation handling, as neurons that die tend to detach and disappear. To examine whether P301S tau+ve neurons with fibrillar tau were dying at a measurable rate in long-term cultures, we labeled neurons with pFTAA and used PI to track cell death in the labeled population over 25 days. Figure 6 shows that identified pFTAA+ve neurons lost plasma membrane integrity and/or disappeared from the cultures over this period while the majority of the unlabeled cells remained PI-negative, except for the occasional appearance of a PI+ve cell, as shown in the image from *t* = 2 days. The number of surviving cells was significantly different (*p* < 0.0144) from day 10 *in vitro* (3 days from the time pFTAA was added) onwards. It is unlikely that pFTAA was toxic, as the percentage of surviving pFTAA-ve cells identified by phase contrast at day 25 was significantly higher than that of pFTAA+ve cells (*p* = 0.0318, paired *t*-test).

## Discussion

Misfolded and hyperphosphorylated tau is now known to be at the fulcrum of many neurodegenerative diseases and it is thus of major interest to understand how it exerts its toxic effects. One of the limitations of current studies aimed at understanding the actions of NFTs (and other abnormally misfolded proteins) in neurodegenerative diseases is the lack of dyes that detect aggregates and fibrils in living neurons. There are many dyes that stain protein preparations of fibrillar tau but few that have been useful for detecting fibrillar tau in living neurons in animal models (Velasco et al., 2008) or in culture (Bandyopadhyay et al., 2007; Lira-De Leon et al., 2013) without toxicity. Kuchibhotla et al. (2014) reported that Methoxy X-04 delivered via the eye vein rapidly detects NFTs in the visual cortex of P301L tau transgenic mice but noted the dye's near-full depletion from the brain within 24 h. Because of its rapid reversibility, they could not corroborate that the dye stained exclusively insoluble NFTs by using antibodies, as they did using the well-established dye Thioflavin S in fixed tissue. We developed the DRG neuron culture system because it can be established from adult mice, the neurons develop pathological forms of tau at the same rate as those that develop in CNS neurons, and some of these properties develop and are maintained during long-term cultures (Mellone et al., 2013).

The present work shows that pFTAA is a non-toxic, high affinity, rapid-acting, and highly specific dye for detection of fibrillar (but not soluble) tau in living neurons *in vivo, ex vivo* and in dissociated cultures of neurons. That the binding of pFTAA is specific to fibrillar tau is shown by the high degree of overlap between pFTAA-labeled neurons and neurons that express the AT100 and MC1 epitopes. Moreover, solubilization of fibrillar tau with formic acid disabled pFTAA binding and MC1 immunolabeling whilst soluble P301S tau detected by HT7 immunolabeling was still abundant in the neurons. pFTAA stained not only the cell bodies of neurons but also the axons, exemplified in the images of intact DRG ganglia (Figure 2A) and in the cultures (Figure 2B), coinciding with immunolabeling for AT100 or MC1.

To our knowledge the affinity of dyes that bind to fibrillar tau in primary neurons has not been reported previously. In neuroblastoma SH-SY5Y cells, uptake and cell viability of the most commonly used pro-aggregating anionic dyes Congo red (CR), Thioflavin S (ThS) and Thiazine Red (TR) were measured after 7 days of treatment (Lira-De Leon et al., 2013). Congo red stained the highest number of cells (55% at 5 μM) and no toxicity was reported. However, EC50 values for ThS and TR were around 75 and 45 μM, respectively, but viability was commensurately reduced even at the lowest concentrations (5 μM). Moreover, these compounds induced aggresome-like structures rather than reporting on their presence, whereas pFTAA is a reporter without evident inducing or dispersing activity. Bandyopadhyay and colleagues (Bandyopadhyay et al., 2007) reported that 10 μM Congo red induced NP40-resistant aggregates in cell pellets from HEK-293T cells expressing human tau, but no uptake of ThS or TR was detected. However, CR also induced considerable toxicity. Due to its high affinity to fibrillar tau, it is tempting to speculate that higher concentrations pFTAA might be used to dissociate NFTs or perturb NFT formation. However, Wegenast-Braun and colleagues showed that repeated weekly intravenous injection of pFTAA at concentrations similar to those used in our study for up to 6 months produced no inhibitory effect on A-beta aggregate formation (Wegenast-Braun et al., 2012). Furthermore, over 25 days *in vitro* we did not observe any instances of living pFTAA+ve neurons that subsequently became pFTAA-ve despite having added a concentration of pFTAA that is 20-fold higher than the half saturation constant of the dye. More work using even higher concentrations will have to be conducted to assess whether pFTAA-bound fibrillar tau remains stable over longer times.

We measured the affinity of pFTAA to fixed DRG neurons from P301S tau mice, finding a half saturation constant of 0.142 μM with a remarkably high correlation coefficient of 0.983 after fitting the data to a binding curve that assumes a single saturable binding site. Even though fibrillar tau may contain more than one binding site per molecule, and several binding sites per cell, it appears that there is no interaction between these individual binding sites (otherwise the curve would show positive or negative cooperativity). It will be interesting to compare this value to that of binding of pFTAA to pure fibrils of tau to determine the relationship between the macroscopic binding to cells and the microscopic binding parameters of the individual fibrils. Unfortunately at present it is not possible to form fibrils of pure tau without resort to fibril-initiating compounds such as polyanions or seeds of fibrillar tau. Hence this data is the first of its kind to probe binding parameters of dyes that are specific to preformed fibrillar tau in neurons.

Along with the relatively high affinity of pFTAA to the fibrils, we found that pFTAA is rapid acting: the shortest time between adding 3 μM pFTAA to the neurons and inspecting them under the microscope was 10 min and already by this time, the intensity of labeling the neurons appeared to be maximal compared to the values obtained 30 min later. Unlike Methoxy X-04 (Kuchibhotla et al., 2014) pFTAA labeling in our mice remained extensive after 48 h and is still detectable 6 months later in fixed brain slices despite extensive washing. Having found that pFTAA labels fibrillar tau in living neurons, we utilized the dye to follow a defined set of neurons over 25 days to examine whether they undergo cell death. We used PI to label dying cells as it is not toxic to living neurons, but because necrotic cells tend to detach from the substrate a short time after they die, we also recorded loss of pFTAA+ve neurons. We found that the rate of cell death appeared to follow two phases; a more rapid phase that lasted over 14 days, and a slower phase that lasted until the end of the study. The reason for this biphasic response is not clear. Since the neurons were cultured for 7 days prior to onset of labeling, it is unlikely that the faster-dying neurons were damaged due to culture preparation. However, unpublished work from our lab indicates that there is high variability in the oxidative status of P301S tau-expressing neurons, which may sensitize a stochastic subset of neurons to death. Nevertheless, it is clear that a sizeable population of the neurons containing fibrillar tau can survive 25 days in culture. Previously, Wegenast-Braun et al. (2012) injected pFTAA to label β-amyloid in APP23 and tau in P301S mice and found no discernable toxicity (Wegenast-Braun et al., 2012). Data presented here corroborates this observation, since several pFTAA+ve neurons survived for 25 day. Additionally, significantly more pFTAA-ve neurons remained viable after 25 days, indicating that the cell death we observed was not due to toxicity but was associated with fibrillar tau. Elucidating the properties of the relatively resistant neurons, which can now be distinguished by using pFTAA, may provide a clue to the factors that are required to resist the possible toxicity of fibrillar tau.

There are some limitations to the use of pFTAA that are worth noting. One proviso was that some neurons appeared to be stained without apparent presence of P301S tau. In some cases, on closer inspection using higher intensity illumination, there was a small remnant of HT7 labeling though the pFTAA still appeared to label a larger part of the neuronal cell body. However, pFTAA also labeled a few *a priori* dead neurons in the culture but these were readily distinguishable under light microscopy as phase dark and grainy. We did not remove the stained dead cells from consideration when we calculated the percentage of pFTAA+ve neurons that were AT100 or MC1+ve, thus accounting for the small percentage of neurons that were recorded as pFTAA+ve but were antibody-negative. Another aspect worth noting is the longevity of pFTAA binding. Although it has been retained without notable fade over several months in the brain sections and ganglia after *in vivo* labeling, in the living cultures, the intensity of pFTAA staining waned over time, though it was still discernable 25 days later. Although we did not replenish the pFTAA in these experiments, adding fresh pFTAA to another set of neurons renewed the staining to the same intensity as that which was recorded after first application. The fade was unlikely to be due to photobleaching, since a continuous 60-min exposure of fixed pFTAA+ve neurons under continuous illumination at the same intensity we use to image pFTAA in the neurons produced minimal loss of fluorescence (see graph here: https://www.dropbox.com/s/1xwyq5bbgoebvmm/pFTAA%20photobleaching.tiff?dl=0). The cumulative intensity x time of exposure in this experiment is 300-fold higher than the intensity the neurons would have been exposed to during the experiment. A more careful quantitative analysis of pFTAA binding intensity together with periodic addition of another vital dye that differentiates neurons with newly-formed fibrillar tau from those stained initially may help inform on the mechanisms of formation and turnover of fibrils in living neurons.

Identifying living neurons containing fibrillar tau with pFTAA opens up many research opportunities. Of utmost interest is the understanding of mechanisms by which neurons with advanced forms of tau pathology die. Although PI labels cells whose plasma membrane is ruptured, the neurons may have followed one of several cell death pathways that culminate in necrosis (Green and Victor, 2012). We can now study these processes by intervening in key steps of each putative death pathway in these identified neurons without having to sample all the neurons and identify those affected after fixation. The strong binding of pFTAA to fibrillar tau over long periods of time coupled to evidence that the dye will dissociate when the fibrils are solubilized will allow us to monitor disaggregation when testing anti-aggregation drugs, and follow at the same time other cellular processes that may participate in the disaggregation process. Likewise, it will be possible to monitor the events leading up to fibrillization as detected by pFTAA and determine the intracellular processes that contribute to the fibrillization process. Finally, we can now purify neurons with fibrillar tau from, for example, the cortex of P301S tau mice, allowing us to develop a biochemical approach to elucidate the mechanisms underlying dysfunction and death of neurons with fibrillar tau. Ultimately, elucidating the events that lead to fibrillar tau formation and neuronal death in our models may have direct relevance to other models of human neurodegenerative diseases, and to the human system in particular.

### Conflict of interest statement

The authors declare that the research was conducted in the absence of any commercial or financial relationships that could be construed as a potential conflict of interest.
